# Targeting the MYC Ubiquitination-Proteasome Degradation Pathway for Cancer Therapy

**DOI:** 10.3389/fonc.2021.679445

**Published:** 2021-06-11

**Authors:** Xiao-Xin Sun, Yanping Li, Rosalie C. Sears, Mu-Shui Dai

**Affiliations:** Department of Molecular & Medical Genetics, School of Medicine and the OHSU Knight Cancer Institute, Oregon Health & Science University, Portland, OR, United States

**Keywords:** MYC, protein stability, ubiquitination, deubiquitination, ubiquitin ligase, deubiquitinating enzyme, SUMOylation, SUMO-specific protease

## Abstract

Deregulated MYC overexpression and activation contributes to tumor growth and progression. Given the short half-life and unstable nature of the MYC protein, it is not surprising that the oncoprotein is highly regulated *via* diverse posttranslational mechanisms. Among them, ubiquitination dynamically controls the levels and activity of MYC during normal cell growth and homeostasis, whereas the disturbance of the ubiquitination/deubiquitination balance enables unwanted MYC stabilization and activation. In addition, MYC is also regulated by SUMOylation which crosstalks with the ubiquitination pathway and controls MYC protein stability and activity. In this mini-review, we will summarize current updates regarding MYC ubiquitination and provide perspectives about these MYC regulators as potential therapeutic targets in cancer.

## Introduction

The c-Myc oncoprotein (MYC thereafter) is a basic helix-loop-helix and leucine zipper (bHLH-LZ) transcription factor that regulates almost all aspects of cell biology by regulating gene transcription, including cell growth and proliferation, apoptosis and senescence, angiogenesis, metabolism, ribosome biogenesis, and stem cell homeostasis (1–4). MYC heterodimerizes with its partner protein MAX *via* its C-terminal bHLH-LZ domain and binds to the E-box element (CACGTG) at target gene promoters (5–7). The N-terminal transcription activation domain (TAD) recruits key transcription co-activators, chromatin modifiers, and mediators to promote the transcription-initiating complex formation and initiate transcription initiation (8, 9). MYC also promotes RNA polymerase II (RNAPII) pause-release upon recruiting the pTEF phosphorylation complex to phosphorylate Serine 2 of the C-terminal domain (CTD) of RNAPII (10). Early individual gene and profiling studies have identified a large number of MYC target genes (4, 11). Recent genome wide studies suggest that MYC might also be a global gene amplifier, promoting the transcription of most, if not all, actively transcribed genes (12–15). Currently, there are several models demonstrating the different modes of MYC transactivation function, including specific-gene regulation, global gene activation, and gene-specific affinity models (1, 3). New studies also suggest that MYC supports genome integrity by clearing stalled RNAPII and resolving transcription-replication conflicts (1, 16).

Since deregulated MYC overexpression contributes significantly to human cancers by regulating the expression of genes involved in almost all aspects of the cancer hallmarks (4, 17, 18), MYC levels and activity must be tightly controlled during normal homeostasis. Under normal physiological condition, MYC is an unstable protein with a half-life less than 15–30 min whereas in growth stimulated cells, MYC is transiently stabilized by shutting down proteasome degradation of MYC. This is controlled by a phosphorylation cascade involving two residues at the TAD: Thr (T) 58 and Ser (S) 62 (19–21). Upon growth stimulation, MYC is phosphorylated by RAS-induced kinases and cyclin-dependent kinases such as CDK2 at S62. Upon reduction in the growth signals, GSK3 is activated to phosphorylate T58, which requires prior S62 phosphorylation. Then, T58 phosphorylation promotes the recruitment of the proline isomerase PIN1 to catalyze the *cis*-to-*trans* isomerization of MYC at Pro (P) 63, followed by the recruitment of the phosphatase PP2A to dephosphorylate MYC at S62. T58 phosphorylated MYC is then targeted by the Fbw7 ubiquitin (Ub) E3 ligase for proteasome degradation (22–25). During the past two decades, more than a dozen ubiquitin ligases have been reported to regulate MYC stability and/or activity. In this mini-review, we briefly describe recent progress in understanding MYC control by the ubiquitin proteasome system including novel ubiquitin ligases and deubiquitinating enzymes and then focus on the perspectives of targeting these molecules for cancer therapy.

## MYC Ubiquitin Ligases

**Figure 1:**
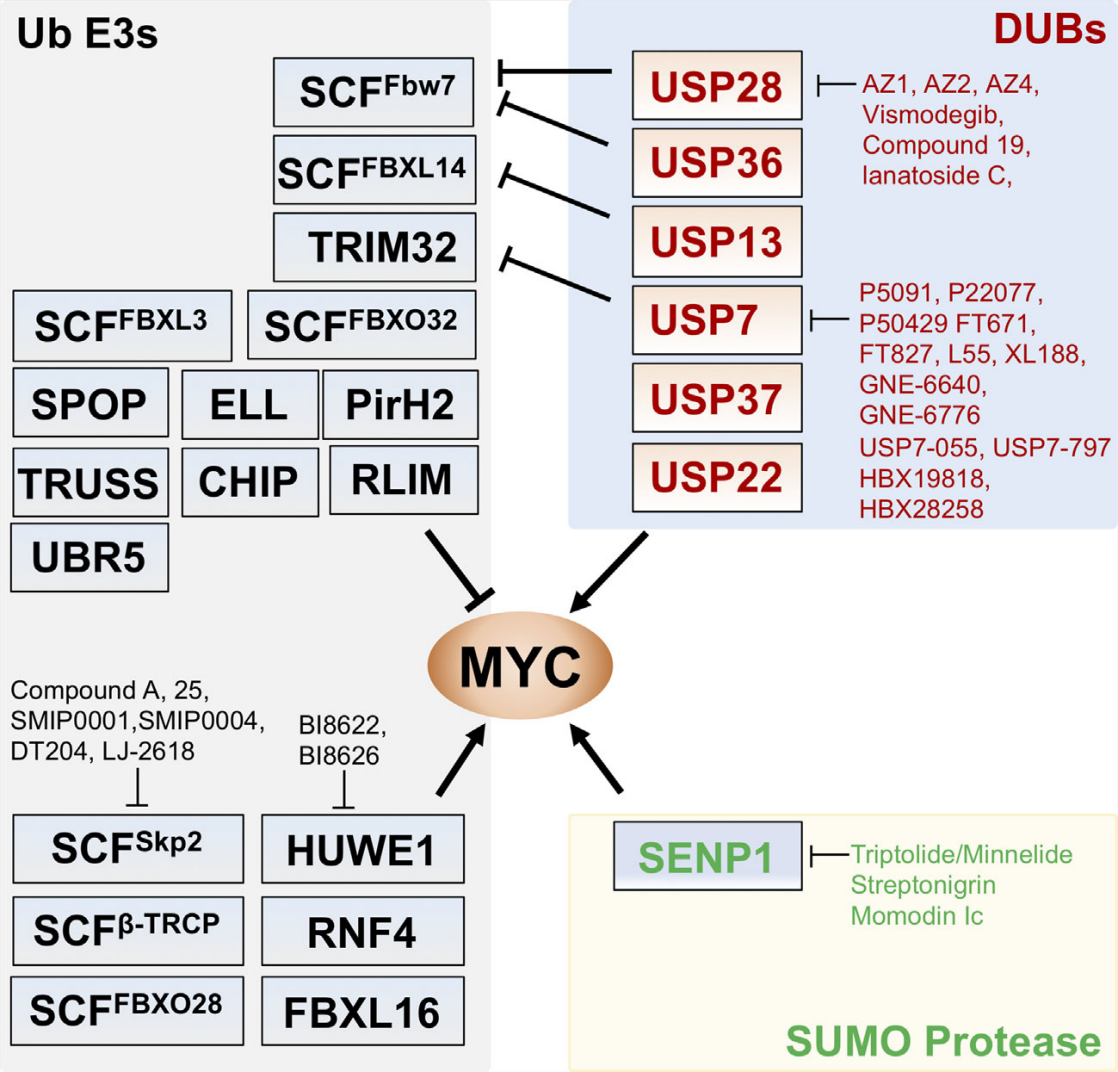
Regulation of MYC by Ub ligases, deubiquitinating enzymes (DUBs), and SUMO protease. Shown are the known Ub ligases (left) mediating MYC ubiquitination, DUBs (upper right) for MYC deubiquitination and SENP1 (lower right) that deSUMOylates and stabilizes MYC. The arrows indicate positive regulation of MYC activity whereas the bars indicate the inhibition. Small molecule inhibitors targeting the indicated Ub ligases, DUBs, and SENP1 are indicated.

To date, at least 18 Ub ligases have been discovered to mediate MYC ubiquitination, which regulates MYC protein stability and/or activity (**Figure 1**). While most of the Ub ligases, such as SCF^Fbw7^, target MYC protein for degradation resulting in the inhibition of MYC activity, several other Ub ligases stabilize MYC. This can be done by antagonizing SCF^Fbw7^-mediated MYC degradation, as in the case for SCF^β-TRCP^, which ubiquitinates MYC *via* K33/K63/K48 mixed linkage, counteracting SCF^Fbw7^-mediated K48-linked MYC ubiquitination and degradation (26). In another example, RNF4, a SUMO-targeted ubiquitin ligase (StubL), mediates K11- and K33-linked ubiquitination of MYC independently of SUMOylation, resulting in MYC stabilization (27). Also, HUWE1 mediates MYC ubiquitination by enhancing the recruitment of p300 and promoting MYC activity without targeting MYC for degradation (28). Yet, HUWE1 ubiquitinates and degrades Miz1, a protein that binds to MYC and accumulates at MYC-bound chromatin associated with repressed transcription (29). Thus, depletion of HUWE1 switches MYC from activating to repressive and suppresses MYC activity (29). On the other hand, SCF^Skp2^-mediated ubiquitination promotes MYC activity, but in this case it is coupled with targeting MYC for proteasome degradation (30, 31). We have reviewed most of the MYC Ub ligases, and the reader can refer to our previous publications (32, 33). Here, we describe newly identified MYC regulators and briefly discuss the role for the MYC ubiquitin ligases in the context of cell cycle and chromatin association.

Recently, the Westermarck group (34) showed that UBR5 ubiquitinates MYC and prevents cells from accumulating excess MYC protein. Interestingly, UBR5 suppresses MYC-dependent priming to therapy-induced apoptosis in cancer cells as it resets MYC to levels that are not enough to induce apoptosis, whereas in normal cells accumulated MYC triggers apoptosis. Indeed, MYC and UBR5 are often co-amplified in MYC-driven human cancers. Yet, UBR5 high expression (UBR5-high) cells dominate MYC-high cells at the single cell level in basal type breast cancers. This study suggests that UBR5-mediated MYC ubiquitination and degradation prevents the accumulation of too much MYC and thus benefits cancer cell survival (34). Therefore, UBR5 may promote tumor cell resistance to cancer therapy. It would be interesting to consider how UBR5 and other regulators of MYC that control MYC ubiquitination levels can tune MYC oncogenic potential.

The leucine-rich repeats (LRR) containing F-box protein FBXL16 has recently been shown to regulate MYC stability by antagonizing SCF^Fbw7^. Interestingly, FBXL16 binds to Skp1 and PP2A but not Cul1, indicating that it may not form a SCF E3 ligase complex (35). FBXL16 binds to wild-type MYC and the T58A or S62A mutant with equal efficiency and does not compete with FBW7 for binding to MYC, but inhibits FBW7-mediated MYC ubiquitination in cells and *in vitro*. As both F-box and the LRR domains are required for FBXL16’s activity to counteract FBW7, it is possible that FBXL16 may form a complex with FBW7 and MYC where it directly suppresses FBW7 ligase activity. On another note, as FBXL16 also binds to PP2A and negatively affects its phosphatase activity (36), it would be interesting to test whether FBXL16 stabilizes MYC partially *via* inhibiting PP2A, thus suppressing MYC S62 dephosphorylation. Nevertheless, this less studied F-box protein functions to promote cancer cell growth and potentiates MYC oncogenic activity.

## MYC Ubiquitination Dynamics

The identification of increasing numbers of Ub ligases targeting MYC suggests that these Ub ligases may control MYC levels and activity dynamically and coordinately and act in a cell context-dependent fashion. For example, FBXO32 is uniquely reported to function under starvation conditions and cell cycle exit, where it catalyzes K48-linked polyubiquitination of MYC at K326 leading to MYC degradation (37, 38). Several other MYC Ub ligases have been reported to function at specific cell cycle phases. TRUSS targets MYC for proteasomal degradation in G1 phase (39, 40). SKP2 expression is high at the G1/S transition and peaks at S phase, and SKP2 promotes S phase entry of MYC containing rat-1 cells, but not MYC null cells (30). In addition, overexpression of SKP2 correlates with the reduction of TRUSS in human cancers (40), suggesting an interplay of MYC Ub ligases in tightly controlling MYC stability during the cell cycle. Also, acting later in the cell cycle, FBXO28 is subjected to regulation by S- and G2/M-phase kinases, cyclin A-CDK2, and cyclin B-CDK1 that mediate phosphorylation of FBXO28 at S344 (41). This activates the Ub ligase activity of FBXO28 to ubiquitinate MYC at C-terminal lysines and recruit p300 co-activator for transactivation of a subset of target genes important for S- and G2/M-phases.

Genome-wide studies revealed wide-spread chromatin binding of MYC, including at high-affinity sites (E-box and CG island regions in the promoter and enhancer regions) bound by endogenous low levels of MYC and at low-affinity sites that are bound by overexpressed and deregulated levels of MYC through a mechanism called “invasion” (13–15, 42). In some experimental conditions, MYC is considered as a transcription amplifier to upregulate most, if not all, active transcribed genes. However, the DNA binding of MYC often does not match its transcription activity (3, 43). Also, MYC binding in certain binding sites with high-affinity promoters, such as ribosome biogenesis genes, can be saturated, and not increase with increasing MYC levels (44). These observations suggest additional control mechanisms between MYC DNA binding and transcriptional regulation.

Emerging evidences suggest that proper chromatin turnover of MYC is critical for its transactivation activity. This is initially observed by two studies showing that SKP2-mediated MYC ubiquitination and proteasome degradation increase MYC activity (30, 31). SKP2 binds to the MYC Box (MB) II which is also the binding motif for the TRAPP co-activator and is essential for MYC transactivation activity. The proteasome can be recruited by MYC and SKP2 to MYC target gene promoters such as the cyclin D2 promoter (31, 45). FBXO28 also binds to MB II and increases MYC activity by non-proteolytic ubiquitination of MYC (41). The role for MYC turnover in target gene transcription activation is further highlighted by a recent study (46) showing that MYC associates with the elongation factor complex PAF1 at promoters and ubiquitination and degradation releases this association. Upon MYC ubiquitination and degradation, the PAF1 complex can then be transferred to and bound to RNA Pol II to promote transcription elongation. Interestingly, PAF1C, a component of the PAF1 complex, interacts with the MBI and phosphorylation at T58 and S62 in MBI promotes PAF1C binding to MYC. Therefore, it would be interesting to examine whether and how MBI phosphorylation coordinates the recruitment of the PAF1 complex with Fbw7-mediated ubiquitination and degradation of MYC, and the role of other E3 ligases such as SKP2 that activate MYC transactivation in this dynamic process controlling transcription. The study also agrees with a recent work by Chen et al. (47) showing that depletion of PAF1 results in an increased release of paused Pol II and transcription elongation of many genes by recruiting the super elongation complex (SEC). Adding to the complexity is that ELL, a component of SEC that promotes transcription elongation, has recently been identified as a MYC Ub ligase. ELL ubiquitinates MYC and targets it for degradation and suppresses MYC-driven tumorigenesis (48). Whether this occurs at the chromatin and how ELL suppresses MYC activity remains to be addressed. Together, these observations are consistent with our studies indicating that MYC DNA binding and turnover are cyclic at promoters together with the cyclic binding of general transcription factors, mediators, and chromatin modifying enzymes as in the case of ERα turnover at promoters (49, 50).

## MYC Deubiquitinating Enzymes (DUBs)

MYC ubiquitination can be removed by deubiquitination. Several deubiquitinating enzymes have been shown to deubiquitinate MYC and regulate its levels and activity. For example, USP28 deubiquitinates MYC *via* counteracting Fbw7α (51, 52), whereas USP36 deubiquitinates MYC in the nucleolus by counteracting Fbw7γ (33, 53). The role of USP36 in regulating MYC ubiquitination and stabilization was also reported in *Drosophila*, showing that the nucleolar isoform of *Drosophila* USP36 (dUSP36) deubiquitinates dMYC and regulates dMYC-dependent cell growth (54). USP22 and USP37 have been shown to deubiquitinate and stabilize MYC (55, 56). USP7 has been shown to deubiquitinate N-Myc (57) and antagonize TRIM32-mediated MYC ubiquitination (58). USP13 can antagonize FBXL14 by removing FBXL14-mediated MYC ubiquitination (59). The Otub6B isoform can regulate MYC levels but not its transcription, although it is unclear whether the isoform can directly mediate MYC deubiquitination (60). While USP36 regulates MYC deubiquitination in the nucleolus, many of the DUBs act on MYC in the nucleoplasm. It is interesting to know whether these DUBs counteract MYC ubiquitin ligases on chromatin to control MYC turnover during transcription and whether they crosstalk with other chromatin modifications, given that USP36 also deubiquitinates H2B (61).

## Crosstalk of MYC SUMOylation With Ubiquitination

Recent studies have shown that MYC is also subject to SUMOylation (62–65). A unique feature about MYC SUMOylation is that it acts promiscuously with respect to the accepting lysines as mutating up to 10 lysines identified by mass spectrometry analysis still failed to abolish MYC SUMOylation (62). This might explain the early observations documenting MYC SUMOylation without a clear effect on MYC stability and activity (63, 65). We recently showed that the SUMO-specific protease SENP1 deSUMOylates MYC (66). Interestingly, SENP1-mediated deSUMOylation stabilizes and activates MYC, likely due to the indirect deubiquitination of MYC as MYC can be co-modified by both Ub and SUMO (66). This is also strongly supported by multiple recent proteomic studies showing ubiquitination of SUMO as well as SUMO-conjugation to multiple lysines of ubiquitin (67, 68). Similar regulation has been observed for HIF1α SUMOylation in that SENP1 deSUMOylation of HIF1α results in HIF1α stabilization as well (69). It is likely that SUMOylation actually regulates the stability of a large number of proteins *via* crosstalk with the Ub system. Nevertheless, the SUMO regulation of MYC adds another layer of complexity to the regulation of MYC protein stability and activity and provides another target in MYC-driven cancer cell growth. Given that SUMOylation plays a key role in transcription regulation (70, 71), it is interesting to understand how MYC SUMOylation/deSUMOylation plays a role in MYC turnover at the chromatin.

## Targeting MYC Ubiquitination Pathway

MYC is commonly considered “undruggable” and direct targeting of MYC is very challenging because of its nucleus localization and the absence of active sites amendable to conventional small molecule ligand-binding (72–74), although this concept has evolved (75, 76). Recently, Omomyc, a peptide that competitively binds to E-box elements as a heterodimer with MAX or as a homodimer and suppresses the binding of MYC to E-boxes (77–79), has been shown to have therapeutic potential *in vivo* in various cancer models (77, 79, 80). Targeting MYC regulatory pathways, such as MYC ubiquitination and deubiquitination whose deregulation contributes to MYC stabilization in cancer, is another highly desirable approach. Several MYC Ub ligases, such as SKP2, HUWE1, and β-TRCP, promote MYC function and thus are promising therapeutic targets (**Figure 1**, **Table 1**). In particular, therapeutically targeting SKP2, which is overexpressed in a variety of cancers, has exciting potential to capitalize on MYC’s pro-apoptotic function. N-terminal ubiquitination of MYC by SKP2 increases MYC’s transactivation of pro-proliferative target genes while loss of SKP2 can not only lead to increased MYC levels but also increased apoptotic activity (30, 31, 107, 108). Inhibitors of SKP2 with anti-tumor properties include several natural products and recently identified small molecule compounds (83, 109). Small molecule compound A was found to inhibit SKP2 incorporation into the SCF complex, thus suppressing its ubiquitin ligase activity (81). SMIP0001 and SMIP0004 (82) have been shown to reduce SKP2 levels and thus induce p27 accumulation. Compound 25 and its analogs (83) disrupt the SKP2-SKP1 interaction in the SCF complex and inhibit the ligase activity and have been shown to suppress cancer cell growth. Chemical library screens identified a novel compound, designated as DT204, that reduces SKP2 binding to Cullin-1 and Commd1, and synergistically enhances BTZ-induced apoptosis (84). NSC689857 and NSC681152 disrupt the SKP2-Cks1 interaction (85), thus inhibiting p27 ubiquitination. It is interesting to examine whether these compounds that specifically inhibit p27 degradation also suppress MYC activity. A selenonucleoside called LJ-2618 downregulates the expression of SKP2 by promoting its degradation and induces G2/M cell cycle arrest in prostate cancer cells and xenograft tumor *in vivo* (86). Likewise, all-*trans* retinoic acid (ATRA) stimulates the ubiquitin-mediated degradation of SKP2 (87). This finding is intriguing because of the known effects of ATRA in stimulating cell differentiation, a consequence of MYC inhibition in some settings (110).

**Table 1:** Inhibitors targeting the MYC degradation pathway.

Name	Mode of action	Clinical trial	References
Compound A	Inhibits SKP2 incorporation into the SCF complex	No	81
SMIP0001, SMIP0004	Reduce SKP2 levels	No	82
Compound 25	disrupts the SKP2-SKP1 interaction	No	83
DT204	Reduces SKP2 binding to Cullin-1 and Commd1	No	84
NSC689857 NSC681152	Disrupt the SKP2-Cks1 interaction	No	85
LJ-2618	Promotes SKP2 degradation	No	86
ATRA	Promotes SKP2 degradation	FDA approved	87
BI8622, BI8626	Inhibit HUWE1	No	29
P5091, P22077, P50429	Covalent USP7 inhibitor	No	88, 89, 115
HBX19818, HBX28258	Covalent USP7 inhibitors	No	90
GNE-6640, GNE-6776	Non-covalent USP7 inhibitors	No	91
FT671	Non-covalent USP7 inhibitor	No	92
FT827, L55	Covalent USP7 inhibitors		93
XL188	Non-covalent USP7 inhibitor	No	94
USP7-055, USP7-797	Non-covalent USP7 inhibitors	No	95
Compound 4	Non-competitive USP7 inhibitor	No	96
Compound 46	USP7 inhibitor	No	97
AZ1, AZ2, AZ4	USP28 inhibitors	No	117
Compound 19	USP28 inhibitor	No	118
Vismodegib	Binds to and inhibits USP28	FDA approved	100
lanatoside C	Inhibits USP28-MYC binding	No	101
Streptonigrin	SENP1 inhibitor	No	102
Triptolide (Minnelide)	SENP1 inhibitor	Phase I and II clinical trials	103, 104
Momordin Ic	SENP1 inhibitor	No	119

Targeting positive ubiquitination mediated by HUWE1 could also suppress MYC’s oncogenic activity. A recent high-throughput screening (HTS) has identified small molecule inhibitors of HUWE1, BI8622 and BI8626, that suppress transactivation of MYC target genes while increasing transrepression and the induction of apoptosis in colorectal cancer cells (29). Treatment of these compounds inhibits MYC-dependent transactivation in colorectal cancer cells but not in stem cells or normal colon epithelial cells, and this effect is associated with the role of HUWE1 inhibition in stabilizing Miz1 and Miz1-mediated suppression of MYC target genes (29). Also, HUWE1 is frequently deregulated in multiple myeloma (MM) and targeting HUWE1 with the small molecule inhibitors in combination with lenalidomide results in synergistic growth inhibition in MM cells *in vitro* and *in vivo* (111).

Many of the MYC DUBs positively regulate MYC stability and activity and could emerge as important cancer therapeutic targets as well. Studies have clearly indicated that different thresholds of MYC elicit different activities and that specific levels of MYC are required to maintain tumorigenesis (112, 113), supporting the idea of dropping MYC levels below these thresholds by strategies like inhibiting DUBs, such as USP28 or USP36 that antagonize FBW7-mediated MYC ubiquitination (52, 53) and USP7 that deubiquitinates N-MYC (57). Indeed, emerging studies have discovered a number of small molecule inhibitors for USP7 (114, 115) (**Table 1**). These USP7 inhibitors include trisubstituted thiophene P5091 (88) and its analogs P22077 (89) and P50429 (90), the acridine derivatives HBX19818 and HBX28258 (91), 2-Amino-4-ethylpyridine derivatives GNE-6640 and GNE-6776 (92), pyrazolo[3,4-d]pyrimidin-4-one-piperidine compounds FT671 and FT827 (93) and the derivative compound L55 (94), a Quinazolin-4-one derivative XL 188 (95), and recently reported novel chemical series including compounds USP7-055 and USP7-797 (96), compound 4 (97), and compound 46 (98). These USP7 inhibitors showed anti-proliferative effect in cancer cell lines and mouse xenograft models. For example, P5091 treatment induced multiple myeloma (MM) cell death, overcomes the resistance to Bortezomib, and inhibits MM xenograft tumor growth (88). Both P22077 and P50429 showed anti-proliferative effect in HCT116 cancer cells and mouse xenograft models (88, 116, 117). Treatment with GNE-6640 and GNE-6776 induced cancer cell death and increases cytotoxicity with chemotherapeutic agents (92). Most of these above studies focused on the role of USP7 in degrading MDM2 to induce p53 and p53-dependent anti-proliferative effects. Yet, effects on p53 mutant cancers are also evident (96). It would be beneficial to understand whether such a role also involves USP7 activity to deubiquitinate N-MYC especially in neuroblastoma with N-MYC amplification or antagonize TRIM32-mediated MYC ubiquitination (58) and whether USP7 deubiquitinates c-MYC as well.

Recently, a high throughput screening (HTS) identified the first set of benzylaminoethanol compounds AZ1, AZ2, and AZ4 that specifically inhibit USP28 (99). By inhibiting USP28, these compounds indeed reduce cellular MYC levels, induce apoptosis, and inhibit cell proliferation in a dose-dependent manner (99). AZ1 was also recently shown to markedly inhibit tumor cell growth and reduce tumor burden in an orthotopically transplanted lung tumor model in mice with well tolerance at doses up to 375 mg/kg (118). Liu et al. (100) reported a new compound 19, a [1,2,3]triazolo[4,5-d] pyrimidine derivative, that specifically inhibits USP28 and reduces gastric cancer cell proliferation and EMT with a better IC_50_ than that of AZ1. These effects are likely due to binding to USP28 and inducing USP28 degradation through ubiquitin-proteasome system (100). Vismodegib, a sonic hedgehog inhibitor used for the treatment of basal cell carcinoma, was recently shown to bind to USP28 and inhibit its DUB activity (101). Vismodegib exhibits selectivity towards USP28 and its evolutionally related USP25. Treatment of cancer cells with Vismodegib reduces the levels of c-Myc and Notch1 and suppresses cell growth (101). In addition, lanatoside C, a cardiac glycoside, has been shown to reduce MYC levels and suppress gastric cancer cell proliferation by inhibiting USP28 binding to MYC, thereby destabilizing MYC, although it remains to be determined whether lanatoside C directly targets USP28 (102).

Given that SENP1 positively regulates MYC levels and activity, SENP1 is an interesting cancer therapeutic target. Several SENP1 inhibitors have been reported (103–105). Streptonigrin (SN), a natural product isolated from Streptomyces flocculus, has been shown to inhibit SENP1 activity (IC_50_ = 0.518 µM towards SENP1, IC_50_ = 6.919 µM towards SENP2) (103). Triptolide, a small natural compound extracted from a Chinese herb Tripterygium wilfordii, has been shown to inhibit SENP1 expression (with IC_50_ = 0.0203 µM in PC-3 cells) (105) and destabilize MYC (104), yet the underlying mechanism is unknown. A water-soluble prodrug of Triptolide called Minnelide has been shown to exhibit promising anti-tumor effects in pancreatic and liver cancers (119). A phase II trial of Minnelide in patients with refractory pancreatic cancer (NCT03117920) was just completed with results pending. Also, a phase I clinical trial of Minnelide (NCT03129139) is ongoing in patients with advanced solid tumors. Also, Momordin Ic (Mc), a natural pentacyclic triterpenic compound, was shown to inhibit SENP1 activity with an IC_50_ of 15.37 µM *in vitro* (106). It is conceivable that by inhibiting MYC deSUMOylation, these SENP1 inhibitors could indirectly suppress MYC deubiquitination, thereby destabilizing MYC and exhibiting anti-proliferative effect in cancer cells.

## Conclusion and Perspectives

Emerging evidence supports the dynamic MYC turnover by proteasome-mediated degradation and the complex crosstalk among different posttranslational modifications (PTMs) of MYC (ubiquitination, phosphorylation, acetylation, SUMOylation, and their reverse processes), resulting in the tight control of MYC transactivation activity, thus emphasizing the importance of MYC in the multi-step regulation of gene transcription and its deregulation in cancer. While several MYC Ub ligases such as the above mentioned SKP2 and HUWE1 have been actively explored as therapeutic targets, small molecule inhibitors suppressing other MYC ligases remain to be identified. This therapeutic strategy holds promise as, for example, knockdown of FBW7 is synthetically lethal to MYC-overexpressing cancer cells (120). Conversely, targeting UBR5 to accumulate MYC beyond the threshold levels that trigger cancer cell apoptosis in MYC-high cancers (34) is another example of inducing MYC synthetic lethality. Together, targeting these MYC posttranslational modifiers could yield potential cancer therapeutics, and additional research understanding the dynamic control processes and the effects of perturbing MYC levels in cancer will be important for their application. Future work would also be needed to further understand the crosstalk between MYC PTMs and the combined intervention of targeting multiple MYC PTMs. Further structure-biology studies and medicinal chemistry optimization could aid in improving target specificity and developing novel compounds. It is hopeful that certain compounds targeting the MYC ubiquitination-proteasome degradation pathways could ultimately move to clinic trials for treating MYC-dependent cancers. If successful, combinational therapies with other targeted therapies could also be desirable to treat advanced cancers, given that MYC crosstalk with many other oncogenic pathways such as RAS, mTOR, HIF signaling, and others.

## Author Contributions

X-XS, YL, RS, M-SD wrote and edited the manuscript. All authors contributed to the article and approved the submitted version.

## Funding

This work was supported by NIH grants R01 CA186241 to M-SD and RS, R01 GM130604 to M-SD, U54 CA209988 to RS, U01 CA224012 to RS, and R01 CA196228 to RS.

## Conflict of Interest

The authors declare that the research was conducted in the absence of any commercial or financial relationships that could be construed as a potential conflict of interest.
